# An Upfront Two-Stent Strategy for True Coronary Bifurcation Lesions with A Large Side Branch in Acute Coronary Syndrome: A Two-Year Follow-Up Study

**DOI:** 10.3390/medicina56030102

**Published:** 2020-02-29

**Authors:** Mustafa Yurtdaş, Ramazan Asoğlu, Mahmut Özdemir, Emin Asoğlu

**Affiliations:** 1 Department of Cardiology, Balıkesir Sevgi Hospital, Paşaalanı Mahallesi, 10020 Balıkesir, Turkey; 2 Department of Cardiology, Adıyaman University Training and Research Hospital, Yunus Emre Mahallesi, 02000 Adıyaman, Turkey; dr.asoglu@yahoo.com; 3 Department of Cardiology, Bayrampasa Kolan Hospital, Terazidere, 34035 Istanbul, Turkey; dr.mahmutozdemr56@yahoo.com; 4 Department of Cardiology, Mardin Community Hospital, Nur Mahallesi, 47100 Mardin, Turkey; asemctf@hotmail.com

**Keywords:** upfront, two-stent strategy, true bifurcation, acute coronary syndrome

## Abstract

*Background and Objectives:* Little is known about the upfront two-stent strategy (U2SS) for true coronary bifurcation lesions (CBLs) in acute coronary syndrome (ACS). We aimed to present our two-year follow-up results on the U2SS by using different two-stent techniques for the true CBL with a large side branch (SB) in ACS patients, including unstable angina (UA), non-ST-segment elevation myocardial infarction (NSTEMI), and ST-segment elevation myocardial infarction (STEMI), and to identify independent predictors of the presence of major adverse cardiac events (MACEs) after intervention. *Materials and Methods:* The study included 201 consecutive ACS patients with true CBLs who underwent percutaneous coronary intervention (PCI) using U2SS from October 2015 to March 2018. Clinical outcomes at follow-up were assessed. MACE was defined as a composite of cardiac death, non-fatal myocardial infarction, and target lesion revascularization (TLR). *Results:* 31.3% of the patients had an UA, 46.3% had an NSTEMI, and 22.4% had an STEMI. CBL was most frequently located in the left anterior descending (LAD)/diagonal artery (59.2%). In total, 71.1% of the patients had a Medina classification (1,1,1). Overall, 62.2% of cases were treated with mini-crush stenting. Clopidogrel was given in 23.9% of the patients; 71.1% of the patients received everolimus eluting stent (EES); and 11.9% received a sirolimus eluting stent (SES). Final kissing balloon inflation was carried out in all patients, with an unsatisfactory rate of 5%. A proximal optimization technique sequence was successfully carried out in all patients. The MACE incidence was 16.9% with a median follow-up period of 2.1 years. There were seven cardiac deaths (3.5%). The TLR rate was 13.4% (*n* = 27), with PCI treatment in 16 patients, and coronary artery bypass grafting treatment in 11 patients. After multivariate penalized logistic regression analysis (Firth logistic regression), clopidogrel use (odds ratio (OR): 2.19; 95% confidence interval (CI): 0.41–2.51; *p* = 0.007) and SES use (OR: 1.86; 95% CI: 0.31–2.64; *p* = 0.014) were independent predictors of the presence of MACE. *Conclusion:* U2SS is feasible and safe for the true CBLs with large and diseased SB in ACS patients, and is related to a relatively low incidence of MACE. Clopidogrel use and SES use may predict the MACE development in ACS patients treated using U2SS.

## 1. Introduction

A coronary bifurcation lesion (CBL) involves coronary artery stenosis adjacent to and/or including the origin of a significant side branch (SB). It is most often haphazardly described based upon the subjective judgement of an interventionalist [1]. Roughly 15%–20% of all percutaneous coronary interventions (PCI) are carried out to treat CBLs, which are commonly encountered by cardiologists in daily practice [1,2]. PCIs for CBLs are associated with a higher incidence of procedural complications, a higher rate of restenosis, and poorer clinical outcomes than non-bifurcation [1]. Although a provisional SB stenting strategy (PSS) for CBL has been proposed, this technique may be associated with the residual ischemia, especially in the true CBL types with a large and diseased SB.

Acute coronary syndrome (ACS) is associated with a high risk for short and long-term mortality [1,3]. There are large ischemic myocardial areas in ACS patients with bifurcation culprit lesions, especially in the presence of a large SB [1]. Therefore, treatment should be directed not only at the main branch (MB), but also at the SB. Although PCI for the true CBL may be difficult, optimal or complete revascularization of jeopardized myocardium by the two-stent strategy (2SS) may be associated with better short- and long-term outcomes in ACS. Little is known about the upfront two-stent strategy (U2SS) for a true CBL in the setting of ACS. In this study, we aimed to present our two-year results on the U2SS by using various two-stent techniques for true CBL with a large SB in ACS patients, including unstable angina (UA), non-ST-elevation myocardial infarction (NSTEMI), and ST-elevation myocardial infarction (STEMI), and to determine independent predictors of major adverse cardiac event (MACE) development after intervention in the univariate and then multivariate analysis.

## 2. Methods

### 2.1. Patient Population

**Figure 1:**
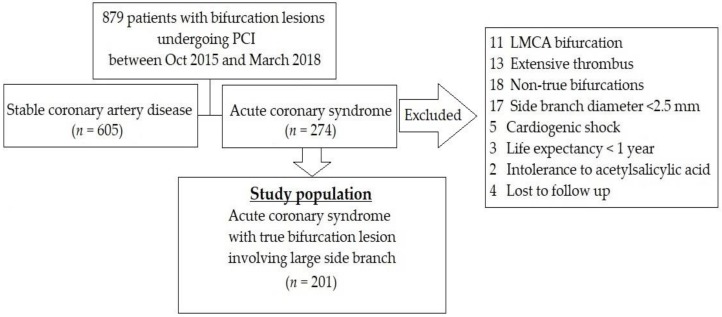
The flow-chart of the study. LMCA: left main coronary artery; PCI: percutaneous coronary intervention.

This study was retrospective. The study population consisted of 201 patients with a true CBL in the setting of ACS, including UA, NSTEMI, and STEMI. From October 2015 to March 2018, all cases without exclusion criteria were enrolled. A true CBL was described as stenosis of > 50% in both the MB and the ostium of the SB according to Medina classification (1,1,1; 1,0,1; 0,1,1) [4] and could be located either in the (1) the left anterior descending (LAD) artery and diagonal branch; (2) the left circumflex artery (LCX) and obtuse marginal branch (OM); or (3) the right coronary artery (RCA), posterior descending artery (PDA), and postero-lateral artery (PLA). The diameters of the MB and the SB by visual estimate were to be ≥ 2.75 mm and ≥ 2.5 mm, respectively. Patients were excluded if they had an extensive thrombus burden on their angiograms, a left main bifurcation lesion, cardiogenic shock, a life expectancy of < 1 year, and/or allergies to any of the drugs (acetylsalicylic acid, clopidogrel, ticagrelor, everolimus, zotarolimus, and sirolimus) used in the study. Patients lost to follow-up were also excluded. The flow-chart of the study is shown in Figure 1. This study was approved by the Ethics Committee (137.A19–21 September 2015), and was carried out in accordance with the Declaration of Helsinki.

### 2.2. Procedures

Coronary procedures were performed using 6 or 7-Fr diagnostic and guiding catheters, through the radial or femoral routes. All patients received loading doses of aspirin (300 mg) and clopidogrel (300 to 600 mg) or ticagrelor (180 mg) before or during the procedure. Dual antiplatelet therapy (DAPT) was given for at least 12 months (acetylsalicylic acid: 100 mg once daily, and clopidogrel: 75 mg once daily, or ticagrelor: 90 mg twice daily). Anticoagulation therapy was given according to the International guidelines [5]. Selections of treatment strategy, stenting technique, and type of drug eluting stent (DES), as well as the decisions to use intravascular ultrasonography (IVUS) and glycoprotein (gp) 2b/3a receptor inhibitors were made at the discretion of the operator.

### 2.3. Bifurcation Stenting Techniques

In the mini-crush stenting technique, both branches were wired and if necessary, pre-dilated. Two stents were placed in the SB and in the MB. The SB stent was pulled back into the MB approximately 1–2 mm and was implanted. After removal of the wire and balloon from the SB, the MB stent was implanted at high pressure, crushing the proximal SB stent. The first proximal optimization technique (POT) was performed. The SB stent was re-wired followed by post-dilatation at high pressure. The procedure was continued with final kissing balloon inflation (FKBI). This was ended by the final (second) POT. In the Culotte stenting technique, both branches were wired and pre-dilated if necessary. First, a stent was deployed across the most angulated branch, usually the SB. Then, the POT was performed. The non-stented branch was then re-wired through the struts of the stent and dilated. A second stent was advanced and expanded into the non-stented branch, usually the MB. Second POT was performed. Wires were exchanged. An FKBI and then final (third) POT were performed. In the V-stenting technique, one stent was advanced in the SB, the other in the MB, and the proximal parts of two stents were positioned to be just abutting each other, creating a new carina (< 5mm) by a simultaneous inflation followed by FKBI. In our study, FKBI was performed in all cases: two non-compliant balloons of diameters equal to the distal MB and SB, respectively, were simultaneously inflated at a low pressure of 10 to 12 atm at the bifurcation after separate high pressure dilation of the SB and the MB. POT was performed to dilate only the part of the stent in the MB just before the carina. POT was performed before and after FKBI in all patients, except those who underwent the V-stenting technique. Unsatisfactory FKBI was defined as the presence of residual stenosis ≥ 20% during FKBI, according to double kissing crush (DKCRUSH) studies [6,7]. Angiographic success was defined as achievement of a thrombolysis in myocardial infarction (TIMI)-3 flow, with a final residual diameter stenosis (DS) < 20% in both MB and SB, and without flow limiting dissection. Procedural success was defined as angiographic success without occurrence of a MACE during the hospital stay.

### 2.4. Quantitative Coronary Angiography

Quantitative coronary angiography (QCA) analysis was performed using offline analysis of the computer-based edge-detection coronary bifurcation system (QAngio XA, version 7.3, Medis, Leiden, The Netherlands). Each bifurcation lesion was viewed in three segments: the proximal and distal main vessels, and the side branch. For quantitative analysis, at least two orthogonal projections were obtained. Minimal lumen diameter (MLD), lumen DS, and reference diameter were measured after the restoration of blood flow in the main and side branches (by guidewire crossing and/or the balloon dilatation) in the STEMI group and before PCI in the non-STEMI and UA groups.

### 2.5. Clinical Follow-Up

Clinical follow-up was carried out with office visits or telephone contacts and completed in 100% of the patients. MACE was defined as a composite of cardiac death, non-fatal myocardial infarction (MI), and target lesion revascularization (TLR). MACE was assessed during in-hospital stays and at a median follow-up period of 2.1 years (interquartile range (IQR): 1.7–2.5) after the intervention. Coronary angiography was performed if anginal symptoms developed and/or myocardial ischemia was detected on non-invasive imaging. Stent thrombosis was classified according to the Academic Research Consortium definitions [8]. In the absence of angiographic evidence, both acute MI associated with the distribution of the coronary artery treated and any death not explained by other reasons were considered as caused by stent thrombosis.

### 2.6. Statistical Analysis

Categorical variables are shown as frequencies and percentage (%) values, and comparisons were made using the chi-square or Fisher’s exact tests. Continuous variables are shown as mean ± SD, and were compared between groups using independent-*t* test or a Mann–Whitney U test, if appropriate. Pearson’s or Spearman correlation analysis was used to detect the relationship between variables. To address concerns about confounding variables (age, diabetes mellitus, clopidogrel use, sirolimus eluting stent (SES) use, renal failure, etc.) that affect MACE development, we conducted a binary logistic regression analysis. All variables with a *p*-value of less than 5% in univariate analysis were entered into multivariate binary logistic regression analysis in order to determine the predictors of the presence of MACE. Because of relatively low incidence of MACE, variables with high odds ratio (OR) and a too wide confidence interval (CI) in the binary logistic regression analysis (diabetes mellitus, clopidogrel use, SES use, Medina (1,1,1), and unsatisfactory final kissing) were entered into the Firth logistic regression analysis (penalized-OR) in order to attenuate their possible inflated effects on MACE development. All probability values were two-sided, and the differences with probability levels (P) of less than 5% were considered as significant. The statistical analyses were done using the SPSS (version 20.0; IBM, SPSS Inc., Chicago, IL, USA) and Stata (version 15.0; StataCorp, Collage Station, TX, USA) software packages.

## 3. Results

A total of 201 ACS patients with true CBL were included in the analysis. Baseline clinical characteristics are shown in Table 1.

Patients had a mean age of 62 ± 10 years, and 37.8% were women, 24.9% had diabetes mellitus. 31.3% of patients had an UA, 46.3% had an NSTEMI, and 22.4% had an STEMI. Angiographic lesion and procedural characteristics of all patients are shown in Table 2.

The true CBL was most frequently located in the LAD/diagonal artery (59.2%), followed by the RCA/PDA (21.4%) and the circumflex (Cx)/obtuse marginalis (OM; 19.4%). In total, 71.1% of the patients had a Medina classification (1,1,1), 12.9% had a Medina classification (1,0,1), and 15.9% had a Medina classification (0,1,1). Angulation of the SB < 70° was seen in 89% of patients. Pre-PCI coronary TIMI-3 flow in the MB and the SB was found in 79% and 86% of patients, respectively. Predilation of the MB and the SB was performed in 67% and 34% of the patients, respectively. The average number of stents used per patient was 2.25 ± 0.5. The mean MB and SB lesion lengths were 22.6 ± 5.7 mm and 11.2 ± 4.0 mm, respectively. The transradial route was able to be used only in 37.3% of patients, since we were unable to cannulate the radial artery due to severe radial artery spasm, especially in female patients (acute coronary syndrome) and rarely anatomical variations, and essentially the absence of radial sheath at the time of the intervention. Angiographic success was 94% based on residual stenosis on SB ostium or MB in 12 patients during the procedure. FKBI was performed in all patients, and unsatisfactory FKBI was observed in 10 (5%) patients. POT sequence was performed in all patients, with success rate of 100%. Procedural success rate was 93.5% based on two in-hospital deaths with angiographic success and one in-hospital death without angiographic success. Overall, 62.2% of patients were treated by the mini-crush, 21.9% by the culotte, and 15.9% by the V-stenting techniques. In case of slow-flow or hazy-image after stenting, a Gp 2b/3a inhibitor was used in 11.4% of the patients. In total, 143 patients (71.1%) received an everolimus eluting stent (EES), 34 patients (16.9%) received a zotarolimus eluting stent (ZES), and 24 patients (11.9%) received a sirolimus eluting stent (SES). Clopidogrel was given in 23.9% of cases, and ticagrelor was given in 76.1% of cases. The mean procedure time was 73 ± 22 minutes. Cumulative MACE rates in hospital and at two years are shown in Table 3.

The cumulative MACE incidence was 16.9% (*n* = 34) at a median follow-up period of 2.1 years. There were seven (3.5%) cardiac deaths: four were sudden cardiac deaths, presumably due to stent thrombosis about 50, 65, and 90 minutes and 14 months after PCI procedure, respectively, and the other three were associated with complications of acute MI which occurred approximately 4, 5, and 9 months after the PCI, respectively. The TLR rate was 13.4% (*n* = 27); 16 patients had repeat PCI and 11 patients underwent coronary artery bypass grafting. The results of QCA analysis for the MB and the SB are shown in Table 4.

After PCI, MLD, and DS improved significantly in both MB and SB. After a median follow-up period of 2.1 years, the clopidogrel use was compared with the ticagrelor use, and SES and ZES uses were compared with the EES use in terms of MACE development, respectively. We did not compare SES use with ZES use. According to univariate analysis, age, ejection fraction, diabetes mellitus, and clopidogrel use (vs ticagrelor use), Medina (1,1,1) classification, MB stent length, the number of stents used per patient, procedural time, and unsatisfactory FKBI and SES use (vs EES use) were significantly associated with the presence of MACE (*p* < 0.05); there was no difference between ZES use and EES use (*p* = 0.532) in terms of MACE development (Table 5).

Multivariate penalized logistic regression analysis (Firth logistic regression) showed that clopidogrel use (OR: 2.19; 95% CI: 0.41–2.51; *p* = 0.007) and SES use (OR: 1.86; 95% CI: 0.31–2.64; *p* = 0.014) were independent predictors of the presence of MACE after the intervention (Table 5).

## 4. Discussion

In this study, our results showed that application of an U2SS for true CBLs with large SB in patients with ACS, including STEMI, was associated with favorable in-hospital and mid-term clinical outcomes.

Most of the studies supporting the PSS have included lesions with trivial or disease-free SB [9,10,11,12,13]. For patients with no to mild ischemia, medical therapy is the treatment of choice and revascularization is associated with increased mortality [14]. For this reason, the PSS can be preferable to a systematic 2SS for lesions with small and/or non-diseased SB. In a study by Cayli et al. [12], the mean SB stenosis was 65% at baseline and 43% post-procedure. In a study of Zhang et al. [13], the mean SB stenosis was 68% at baseline, 48% post-procedure, and 63% at 9 months. These data suggest that the jailed-wire, jailed-balloon, or its modified techniques do not exactly improve SB disease, indicating that myocardium supplied by the diseased SB still remains ischemic (residual ischemia), which is associated with adverse clinical outcomes. Recently, in a study of CBL undergoing PSS, Hakim et al. reported that despite the presence of TIMI-3 flow in the SB after only MB stenting and POT, SB fractional flow reserve was less than 0.75 in 30% of patients, which improved to more than 0.75 after SB dilation or stenting plus final POT [15]. Their findings suggest that if true CBL with large SB was treated using U2SS, the rate of residual ischemia would be reduced. Similar to our findings, the mean SB stenosis was 68% at baseline, and it was 19% post-procedure in a study of true CBL with large SB treated by U2SS [16]. In our study, all lesions were true CBLs with a large SB of 2.7 mm in mean diameter. The mean SB stenosis was 74% at baseline, and it was 5.7% post-procedure with TIMI-3 flow, suggesting that there were no ischemic myocardial areas. Complex CBLs with large SB with significant ostial disease are best treated with an U2SS based on expert consensus opinion [1]. The European Bifurcation Coronary TWO (EBC-TWO) and the Nordic-Baltic Bifurcation Study-IV studies could not show any significant difference in MACE rate at follow-up in the 2SS when compared to the PSS [17,18]. However, in the DKCRUSH-II, the 2SS was found to be associated with improved outcomes compared with the PSS [7]. The DKCRUSH-V found that the U2SS resulted in a lower MI, stent thrombosis, and TLR compared with the PSS [19].

The number of studies pertaining to bifurcation stenting in STEMI is very scant. A sub-study of the DKCRUSH-II showed that both stenting strategies in patients with STEMI had similar immediate and mid-term clinical outcomes [20]. In contrast, a sub-study of the Korean Coronary Bifurcation Study (COBIS)-2 showed that the 2SS had significantly higher rates of MACE than the PSS in STEMI [21]. The rate of STEMI patients in our study was 22.4%, a higher percentage than in previous studies (13.5% and 12.7%), and none of our patients received thrombolytic therapy. We detected lower rates of MACE in our STEMI patients (15.6%) than those in the DKCRUSH-II (21%) and the COBIS-2 (29%) studies. The most probable reasons for the differences are lesion complexity, the application of FKBI and POT, the type of stents and anti-aggregants used, and the time from symptom onset to PCI. The time from symptom onset to PCI was less than 12 h in a sub-study of the DKCRUSH-II, it was less than 4 hours in our study, and it was not mentioned in the COBIS-2. The POT was not performed in the DKCRUSH-II nor in the COBIS-2, in contrast to our study.

Our favorable results can be explained in several ways: First, “bail-out” SB stent through the MB stent struts may be difficult and/or culminate in incomplete expansion or edge dissections. “Bail-out” stenting is one of the independent predictors of mid-term MACE after PCI for CBLs [22]. Therefore, we planned and performed the U2SS for true CBL in all ACS patients. Second, failure to apply FKBI is related to both a higher rate of stent thrombosis and a greater rate of restenosis, TLR, and MACE [1,23]. Additionally, the POT facilitates optimal guidewire re-crossing following stenting, corrects the asymmetric deployment of the stent related to FKBI, and prevents both strut malaposition and stent underexpansion, which are closely associated with neoatherosclerosis and thus adverse events [1,24,25]. Clinical and bench studies have shown the importance of the POT after FKBI to improve the results [1,24,25,26]. Although the double kissing (DK) strategy of the DK-crush technique has been reported to be superior to other techniques, the POT has not been applied in the DKCRUSH studies [6,7,27], except for the DKCRUSH-V [19]. In our study, we did not perform the double kissing strategy. We performed FKBI in all patients with a satisfactory rate of 95%. Additionally, we routinely performed the POT sequence in the mini-crush and culotte techniques. Our FKBI success (95%) was higher than in the formerly reported trials of complex stenting techniques, in which rates of kissing balloon success were nearly 75% [6,9]. The most probable reasons for the high rate of success in our study are the routine POT application, large SB diameter, bifurcation angle of < 70° (in 89% of our patients), and the expertise and experience of the operator in performing bifurcation treatment. In line with our findings, Zhang et al. performed the FKBI and then the POT in all true bifurcation lesions [16]. Third, the new-generation DES is superior to the early-generation DES in terms of favorable clinical results in bifurcation lesions [28]. In true CBL treated by the 2SS, the COBIS-2 showed that EES was superior to SES [29]. EES and ZES have similar efficacy and safety in ACS and bifurcation lesions [30]. Interestingly, we found that SES use was one of the important predictors of MACE after the procedure. Neoatherosclerosis, as one of the possible causes of the stent failure and adverse events, might take place more commonly in SES use than in EES use, which may have resulted from the both chemical and physical properties of SES [25,29]. Lastly, ticagrelor has superior outcomes than clopidogrel in ACS [31]. Interestingly, in almost all bifurcation studies, ACS patients were given clopidogrel. In our study, clopidogel was given to 24% of patients and found to be another important predictor of the presence of MACE. In harmony with our finding, Zheng et al. found that for patients with bifurcation stenting, ticagrelor was superior to clopidogrel in terms of MACE and MI rates [32].

### Limitations

There are several limitations to our study. First, it is an observational and single-arm design with a limited sample size. The selection of treatment strategy, stent type, and stenting technique and the decision to use IVUS and medications were left to the operator’s discretion. Second, owing to the retrospective design, follow-up angiograms were performed only in patients with symptoms and/or ischemia on non-invasive imaging. For this reason, clinical follow-up alone (with the office visits and/or telephone contacts) might not be enough to identify all relevant clinical events accurately. Finally, IVUS was not used routinely and optical coherence tomography was not performed.

## 5. Conclusions

The use of an U2SS for true CBLs with large SBs in patients presenting with ACS including STEMI is safe and feasible, with relatively low incidence of MACE during a follow-up period of 2.1 years. The U2SS may be considered in true CBLs with large and diseased SBs in all ACS patients. Clopidogrel use and SES use may predict MACE development in this patient group after the intervention with U2SS.

## Figures and Tables

**Table 1:** Baseline clinical characteristics of all patients (*n* = 201).

Variables	
Age (years), mean ± SD	62 ± 10
Sex (female) *n*, (%)	76 (37.8)
Hypertension *n*, (%)	111 (55.2)
Diabetes mellitus *n*, (%)	50 (24.9)
Hypercholesterolemia *n*, (%)	90 (44.8)
Family history *n*, (%)	59 (29.4)
Smoking status *n*, (%)	47 (23.4)
Ejection fraction, (%)	51 ± 8
Prior percutaneous coronary intervention *n*, (%)	25 (12.4)
Prior coronary surgery *n*, (%)	9 (4.5)
Chronic kidney disease *n*, (%)	11 (5.5)
Clinical presentation *n*, (%)	
Unstable angina pectoris *n*, (%)	63 (31.3)
Non-ST-elevation myocardial infarction *n*, (%)	93 (46.3)
ST-elevation myocardial infarction *n*, (%)	45 (22.4)

**Table 2:** Angiographic lesion and procedural features of all patients (*n* = 201).

Variables	
Lesion location, *n* (%)	
LAD-Diagonal	119 (59.2)
LCx-OM	39 (19.4)
RPDA-RPLA	43 (21.4)
Medina classification, *n* (%)	
1,1,1	143 (71.1)
1,0,1	26 (12.9)
0,1,1	32 (15.9)
Bifurcation angle, *n* (%)	
<70°	179 (89)
≥70°	22 (11)
Pre-PCI coronary TIMI-3 flow, *n* (%)	
Main branch	159 (79)
Side branch	173 (86)
Two-stent techniques, *n* (%)	
Mini-crush	125 (62.2)
Culotte	44 (21.9)
V-stenting	32 (15.9)
Transradial approach, *n* (%)	75 (37.3)
Glycoprotein 2b/3a inhibitors, *n* (%)	23 (11.4)
The number of stents used per patient	2.25 ± 0.5
Lesion and stent lenghts, (mm)	
Main branch lesion lenght	22.6 ± 5.7
Main branch stent lenght	26.4 ± 6.5
Side branch lesion lenght	11.2 ± 4.0
Side branch stent lenght	15.0 ± 4.3
Stent types, *n* (%)	
Everolimus eluting stent	143 (71.1)
Zotarolimus eluting stent	34 (16.9)
Sirolimus eluting stent	24 (11.9)
Antiplatelet therapy, *n* (%)	
Clopidogrel	48 (23.9)
Ticagrelor	153 (76.1)
Intravascular ultrasonography use, *n* (%)	83 (41)
Procedural features, *n* (%)	
Predilation in main branch	135 (67)
Predilation in side branch	69 (34)
Angiographic success	189 (94)
Procedural success	188 (93.5)
Unsatisfactory final kissing balloon inflation	10 (5)
Procedure time (min), mean ± SD	73 ± 22
Follow-up (years), median (IQR)	2.1 (1.7–2.5)

IQR: interquartile range; LAD: left anterior descending; LCx: left circumflex; OM: obtuse marginalis; PCI: percutaneous coronary intervention; RPDA: right posterior descending artery; RPLA: right posterior lateral artery; TIMI: thrombolysis in myocardial infarction.

**Table 3:** Major adverse cardiac event (MACE) rates in hospital and at two years in the study population (*n* = 201).

Variables	*n* (%)
(A) MACE rates in hospital	3 (1.5)
(1) Non-fatal myocardial infarction	0 (0.0)
(2) Cardiac death	3 (1.5)
(3) Target lesion revascularization	0 (0.0)
(B) Cumulative MACE rates at 2 years	34 (16.9)
(1) Non-fatal myocardial infarction	3 (1.5)
(2) Cardiac death	7 (3.5)
(3) Target lesion revascularization	27 (13.4)
(3a) Percutaneous coronary intervention	16 (8.0)
(3b) Coronary artery bypass grafting	11 (5.5)

**Table 4:** Pre- and post-procedure quantitative coronary angiographic analyses for main branch and side branch in all study population (*n* = 201).

	Pre-Procedure	Post-Procedure	*p*-Value
**Proximal main branch**			
Reference vessel diameter (mm)	3.10 ± 0.38	3.11 ± 0.38	0.071
Minimum lumen diameter (mm)	0.67 ± 0.32	2.97 ± 0.36	<0.001
Diameter stenosis, %	78 ± 9.7	4.4 ± 3.3	<0.001
**Distal main branch**			
Reference vessel diameter (mm)	2.91 ± 0.34	2.92 ± 0.33	0.160
Minimum lumen diameter (mm)	0.61 ± 0.23	2.77 ± 0.36	<0.001
Diameter stenosis, %	79 ± 7.2	5.5 ± 2.3	<0.001
**Side branch**			
Reference vessel diameter (mm)	2.7 ± 0.22	2.7 ± 0.22	0.575
Minimum lumen diameter (mm)	0.69 ± 0.24	2.53 ± 0.26	<0.001
Diameter stenosis, %	74 ± 8.6	5.7 ± 5.6	<0.001

**Table 5:** Univariate and multivariate analysis to determine predictors of the presence of major adverse cardiac events (MACEs) in the study population.

Variables	Univariate Analysis			Multivariate Analysis		
	OR	95% CI	*p*-Value	OR	95% CI	*p*-Value
Clinical parameters						
Age (mean, years)	1.06	1.02–1.09	0.004	1.04	0.99-1.10	0.095
Sex (female)	1.57	0.70–3.49	0.270			
Hypertension	1.38	0.65–2.94	0.401			
Diabetes mellitus	2.18	0.99–4.75	0.048	2.35	0.75-7.30	0.139
				1.85 *	−0.057–2.03	0.166
Hypercholesterolemia	1.11	0.52–2.34	0.769			
Ejection fraction	0.94	0.89–0.99	0.019	0.96	0.91–1.02	0.228
Chronic kidney disease	0.98	0.91–3.67	0.881			
Unstable angina	1.24	0.57–2.69	0.586			
NSTEMI	0.90	0.43–1.89	0.783			
STEMI	0.88	0.35–2.18	0.782			
Clopidogrel (vs ticagrelor)	11.8	5.15–27.36	<0.001	5.48	1.68–17.94	0.005
				2.19 *	0.41–2.51	0.007
Anatomic, angiographic and procedural parameters						
LAD-Diagonal	0.55	0.26–1.16	0.117			
LCx-OM	1.98	0.85–4.59	0.110			
RPDA-RPLA	1.16	0.48–2.78	0.739			
Medina (1,1,1)	2.69	0.98–7.35	0.046	2.72	0.72–10.26	0.140
				1.41 *	−0.33–2.11	0.158
Medina (1,0,1)	0.60	0.17–2.14	0.437			
Medina (0,1,1)	0.28	0.06–1.25	0.097			
Mini-Crush	1.56	0.70–3.49	0.270			
Culotte	0.91	0.36–2.25	0.840			
V-stenting	0.46	0.13–1.61	0.224			
The number of stents	3.02	1.61–5.66	0.001	1.72	0.57–5.20	0.333
ZES (vs everolimus)	1.34	0.53–3.40	0.532			
SES (vs everolimus)	17.67	6.64–47.01	<0.001	5.02	1.34–18.81	0.017
				1.86 *	0.31–2.64	0.014
MB stent length	1.10	1.04–1.17	0.001	1.19	0.71–2.01	0.499
MB proximal-MLD (bp)	2.23	0.72–6.92	0.163			
MB distal-MLD (bp)	1.32	0.27–6.29	0.728			
MB proximal-MLD (ap)	1.61	0.61–4.24	0.333			
MB distal-MLD (ap)	1.40	0.54–5.31	0.107			
Side branch lesion length	1.07	0.97–1.17	0.164			
Side branch stent length	1.06	0.98–1.16	0.148			
Side branch MLD (bp)	2.00	0.46–8.70	0.356			
Side branch MLD (ap)	3.06	0.81–11.63	0.100			
Procedure time	1.02	1.00–1.04	0.019	1.02	0.99–1.04	0.175
Unsatisfactory final kissing	5.59	1.52–20.52	0.010	4.77	0.86–26.45	0.074
				1.71 *	−0.21–3.02	0.087

* After the Firth logistic regression analysis. ap: after the procedure; bp: before the procedure; CI: confidence interval; LAD: left anterior descending; LCx: left circumflex; OM: obtuse marginalis; MB: main branch; MLD: minimal lumen diameter; NSTEMI: non-ST-elevation myocardial infarction; OR: odds ratio; RPDA: right posterior descending artery; RPLA: right posterior lateral artery; SES: sirolimus-eluting stent; STEMI: ST-elevation myocardial infarction; ZES: zotarolimus-eluting stent.

## References

[1] Lassen J.F., Holm N.R., Banning A., Burzotta F., Lefèvre T., Chieffo A., Hildick-Smith D., Louvard Y., Stankovic G. (2016). Percutaneous coronary intervention for coronary bifurcation disease: 11th consensus document from the European Bifurcation Club. EuroIntervention.

[2] Myler R.K., Shaw R.E., Stertzer S.H., Hecht H.S., Ryan C., Rosenblum J., Cumberland D.C., Murphy M.C., Hansell H.N., Hidalgo B. (1992). Lesion morphology and coronary angioplasty: Current experience and analysis. J. Am. Coll. Cardiol..

[3] Alcock R., Yong A., Ng A., Chow V., Cheruvu C., Aliprandi-Costa B., Lowe H., Kritharides L., Brieger D. (2013). Acute coronary syndrome and stable coronary artery disease: Are they so different? Long-term outcomes in a contemporary PCI cohort. Int. J. Cardiol..

[4] Medina A., Suárez de Lezo J., Pan M. (2006). A new classification of coronary bifurcation lesions. Rev. Esp. Cardiol. Engl. Ed..

[5] Neumann F.-J., Sousa-Uva M., Ahlsson A., Alfonso F., Banning A.P., Benedetto U., Byrne R.A., Collet J.-P., Falk V., Head S.J. (2019). 2018 ESC/EACTS guidelines on myocardial revascularization. Eur. Heart J..

[6] Chen S., Zhang J., Ye F., Chen Y., Patel T., Kawajiri K., Lee M., Kwan T., Mintz G., Tan H. (2008). Study comparing the double kissing (DK) crush with classical crush for the treatment of coronary bifurcation lesions: The DKCRUSH-1 bifurcation study with drug-eluting stents. Eur. J. Clin. Investig..

[7] Chen S.-L., Santoso T., Zhang J.-J., Ye F., Xu Y.-W., Fu Q., Kan J., Zhang F.-F., Zhou Y., Xie D.-J. (2017). Clinical outcome of double kissing crush versus provisional stenting of coronary artery bifurcation lesions: The 5-year follow-up results from a randomized and multicenter DKCRUSH-II study (Randomized Study on Double Kissing Crush Technique Versus Provisional Stenting Technique for Coronary Artery Bifurcation Lesions). Circ. Cardiovasc. Interv..

[8] Cutlip D.E., Windecker S., Mehran R., Boam A., Cohen D.J., van Es G.-A., Gabriel Steg P., Morel M., Mauri L., Vranckx P. (2007). Clinical end points in coronary stent trials: A case for standardized definitions. Circulation.

[9] Behan M.W., Holm N.R., de Belder A.J., Cockburn J., Erglis A., Curzen N.P., Niemelä M., Oldroyd K.G., Kervinen K., Kumsars I. (2016). Coronary bifurcation lesions treated with simple or complex stenting: 5-year survival from patient-level pooled analysis of the Nordic Bifurcation Study and the British Bifurcation Coronary Study. Eur. Heart J..

[10] Gwon H.-C., Choi S.-H., Song Y.B., Hahn J.-Y., Jeong M.-H., Seong I.-W., Kim H.-S., Rha S.W., Yang J.-Y., Yoon J.H. (2010). Long-term clinical results and predictors of adverse outcomes after drug-eluting stent implantation for bifurcation lesions in a real-world practice: The COBIS (Coronary Bifurcation Stenting) registry. Circ. J. Off. J. Jpn. Circ. Soc..

[11] Hildick-Smith D., de Belder A.J., Cooter N., Curzen N.P., Clayton T.C., Oldroyd K.G., Bennett L., Holmberg S., Cotton J.M., Glennon P.E. (2010). Randomized trial of simple versus complex drug-eluting stenting for bifurcation lesions: The British Bifurcation Coronary Study: Old, new, and evolving strategies. Circulation.

[12] Çaylı M., Şeker T., Gür M., Elbasan Z., Şahin D.Y., Elbey M.A., Çil H. (2015). A Novel-Modified Provisional Bifurcation Stenting Technique: Jailed Semi-Inflated Balloon Technique. J. Intervent. Cardiol..

[13] Zhang W., Ji F., Yu X., Wang X. (2019). Long-term treatment effect and adverse events of a modified jailed-balloon technique for side branch protection in patients with coronary bifurcation lesions. BMC Cardiovasc. Disord..

[14] Iwasaki K. (2014). Myocardial ischemia is a key factor in the management of stable coronary artery disease. World J. Cardiol..

[15] Hakim D., Chatterjee A., Alli O., Turner J., Sattar A., Foin N., Leesar M.A. (2017). Role of proximal optimization technique guided by intravascular ultrasound on stent expansion, stent symmetry index, and side-branch hemodynamics in patients with coronary bifurcation lesions. Circ. Cardiovasc. Interv..

[16] Zhang D., He Y., Yan R., Yin D., Feng L., Xu B., Yang Y., Zhu C., Dou K. (2019). A novel technique for coronary bifurcation intervention: Double rewire crush technique and its clinical outcomes after 2 years of follow-up. Catheter. Cardiovasc. Interv..

[17] Hildick-Smith D., Behan M.W., Lassen J.F., Chieffo A., Lefèvre T., Stankovic G., Burzotta F., Pan M., Ferenc M., Bennett L. (2016). The EBC TWO Study (European Bifurcation Coronary TWO) A Randomized Comparison of Provisional T-Stenting Versus a Systematic 2 Stent Culotte Strategy in Large Caliber True Bifurcations. Circ. Cardiovasc. Interv..

[18] Kumsars P.I. Two-year results in the Nordic-Baltic bifurcation study IV. https://www.tctmd.com/slide/two-year-results-nordic-baltic-bifurcation-study-iv.

[19] Chen X., Li X., Zhang J.-J., Han Y., Kan J., Chen L., Qiu C., Santoso T., Paiboon C., Kwan T.W. (2019). 3-Year outcomes of the DKCRUSH-V trial comparing DK crush with provisional stenting for left main bifurcation lesions. JACC Cardiovasc. Interv..

[20] Kwan T.W., Gujja K., Liou M.C., Huang Y., Wong S., Coppola J., Chen S. (2013). Bifurcation stenting in patients with ST-Segment elevation myocardial infarction: An analysis from dkcrush II randomized study. Catheter. Cardiovasc. Interv..

[21] Choi K.H., Song Y.B., Jeong J.-O., Park T.K., Lee J.M., Yang J.H., Hahn J.-Y., Choi S.-H., Choi J.-H., Lee S.H. (2018). Treatment strategy for STEMI with bifurcation culprit lesion undergoing primary PCI: The COBIS II Registry. Rev. Esp. Cardiol. Engl. Ed..

[22] Zimarino M., Briguori C., Amat-Santos I.J., Radico F., Barbato E., Chieffo A., Cirillo P., Costa R.A., Erglis A., Gamra H. (2019). Mid-term outcomes after percutaneous interventions in coronary bifurcations. Int. J. Cardiol..

[23] Sgueglia G.A., Chevalier B. (2012). Kissing balloon inflation in percutaneous coronary interventions. JACC Cardiovasc. Interv..

[24] Hoye A. (2017). The proximal optimisation technique for intervention of coronary bifurcations. Interv. Cardiol. Rev..

[25] Borovac J.A., D’Amario D., Vergallo R., Porto I., Bisignani A., Galli M., Annibali G., Montone R.A., Leone A.M., Niccoli G. (2019). Neoatherosclerosis after drug-eluting stent: A novel clinical and therapeutic challenge. Eur. Heart. J. Cardiovasc. Pharmacother..

[26] Finet G., Derimay F., Motreff P., Guerin P., Pilet P., Ohayon J., Darremont O., Rioufol G. (2015). Comparative analysis of sequential proximal optimizing technique versus kissing balloon inflation technique in provisional bifurcation stenting: Fractal coronary bifurcation bench test. JACC Cardiovasc. Interv..

[27] Chen S.-L., Xu B., Han Y.-L., Sheiban I., Zhang J.-J., Ye F., Kwan T.W., Paiboon C., Zhou Y.-J., Lv S.-Z. (2015). Clinical outcome after DK crush versus culotte stenting of distal left main bifurcation lesions: The 3-year follow-up results of the DKCRUSH-III study. JACC Cardiovasc. Interv..

[28] Navarese E.P., Kowalewski M., Kandzari D., Lansky A., Górny B., Kołtowski Ł., Waksman R., Berti S., Musumeci G., Limbruno U. (2014). First-generation versus second-generation drug-eluting stents in current clinical practice: Updated evidence from a comprehensive meta-analysis of randomised clinical trials comprising 31 379 patients. Open Heart.

[29] Cho Y., Koo B.-K., Song Y.B., Hahn J.-Y., Choi S.-H., Gwon H.-C., Rha S.W., Yu C.W., Park J.-S., Bae J.-H. (2015). Comparison of the first-and second-generation limus-eluting stents for bifurcation lesions from a Korean multicenter registry. Circ. J..

[30] Iqbal J., Serruys P.W., Silber S., Kelbaek H., Richardt G., Morel M.-A., Negoita M., Buszman P.E., Windecker S. (2015). Comparison of zotarolimus-and everolimus-eluting coronary stents: Final 5-year report of the RESOLUTE all-comers trial. Circ. Cardiovasc. Interv..

[31] Wallentin L., Becker R.C., Budaj A., Cannon C.P., Emanuelsson H., Held C., Horrow J., Husted S., James S., Katus H. (2009). Ticagrelor versus clopidogrel in patients with acute coronary syndromes. N. Engl. J. Med..

[32] Zheng W., Li Y., Tian J., Li L., Xie L., Mao Q., Tong W., Zhou D., Azzalini L., Zhao X. (2019). Effects of Ticagrelor versus Clopidogrel in Patients with Coronary Bifurcation Lesions Undergoing Percutaneous Coronary Intervention. BioMed Res. Int..

